# Professional Responsibility Governed by a Risk‐Oriented Care Logic: Nursing Staff's Experiences of Caring for Persons With Anorexia Nervosa in General Psychiatric Inpatient Care

**DOI:** 10.1111/inm.70297

**Published:** 2026-06-22

**Authors:** Anna Sandsten, Maria Strömbäck, Sebastian Gabrielsson, Git‐Marie Ejneborn Looi, Britt‐Marie Lindgren

**Affiliations:** ^1^ Department of Nursing and Umeå Centre for Gender Studies, Gender Research School Umeå University Umeå Sweden; ^2^ Department of Community Medicine and Rehabilitation Umeå University Umeå Sweden; ^3^ Department of Clinical Science Umeå University Umeå Sweden; ^4^ Department of Health, Education and Technology Luleå University of Technology Luleå Sweden; ^5^ Department of Nursing Umeå University Umeå Sweden

**Keywords:** anorexia nervosa, experiences, nursing staff, psychiatric inpatient care, qualitative

## Abstract

Mental health nursing in inpatient psychiatry is shaped by control and standardisation, yet little is known about how these conditions are sustained in everyday care for persons with anorexia nervosa. This study aimed to explore how nursing staff experience and understand care for persons with anorexia nervosa within general psychiatric inpatient care settings, with particular attention to the conditions shaping what comes to count as legitimate nursing practice. Nine nursing staff with experience caring for persons with anorexia nervosa in general psychiatric inpatient care in Sweden participated in the study. Data were collected in 2025 using semi‐structured qualitative interviews and were subjected to qualitative content analysis with a Foucauldian‐inspired abductive approach. Findings are presented as two themes, *Nursing practice governed by a risk‐oriented care logic* and *Responsibility as a burden and a possibility within nursing practices.* Within those themes are five sub‐themes, *Making sense of AN through risk and stabilisation; Enacting control through routines and treatment plans; Normalising coercion in everyday care; Navigating responsibility under unstable care conditions* and *Producing alternative care practices through relational work*. Findings suggest that discourses of risk and stabilisation organise nursing practice through a risk‐oriented care logic that shapes what is recognised as legitimate care and professional competence. Within these conditions, relational and individualised nursing practices become difficult to sustain while responsibility and coercive measures are framed as necessary responses to risk. This study is reported in accordance with the COREQ guidelines.

## 1 Introduction

Mental health nursing is largely enacted as relational work within psychiatric inpatient care contexts that are characterised by control, standardisation and ethical tension (Gabrielsson et al. 2016; Molin et al. 2016; Söderberg et al. 2021; Zugai et al. 2019). Despite extensive documentation of these conditions, little is known about how and why they are produced and sustained in everyday practice, particularly, in nursing care for persons diagnosed with anorexia nervosa (AN) in general psychiatric inpatient care (GPIC) settings. A closer examination of nursing staff's experiences of caring for persons with AN in GPIC may offer insights into how prevailing ways of understanding illness, care and professional responsibility shape everyday mental health nursing.

## 2 Background

Research on AN is largely oriented towards measurable clinical outcomes such as body mass index (BMI), weight restoration and medical complications, as well as towards specific treatment interventions including meal support, nasogastric feeding and structured treatment programmes (Sandsten, Lindgren, et al. 2025). More recent discussions have problematised the notion of treatment‐resistant AN (Elwyn 2023). Research has predominantly been conducted in the context of specialised eating disorder services (Sandsten, Lindgren, et al. 2025). However, this focus contrasts with clinical practice, as a considerable proportion of persons with AN receive GPIC (Toppino et al. 2024).

Persons with AN may require admission to GPIC for a variety of reasons, such as medical instability due to low weight, insufficient long‐term improvement from outpatient treatment, suicidal risk or presence of comorbid conditions that complicate outpatient care (Rankin et al. 2023; Toppino et al. 2024). GPIC units admit diagnostically heterogeneous populations, which implies variable staff exposure to specific conditions including AN (Calhoun et al. 2022; Toppino et al. 2024).

Nursing staff in GPIC are professionals with various formal qualifications, who share responsibility for patients' mental and physical health. Registered nurses have the nursing responsibility and coordinate care interventions, while psychiatric specialist nurses contribute advanced clinical knowledge (Salberg et al. 2019). However, much of the continuous patient contact, including observation and relational support, is carried out by nursing assistants who work most closely to persons in care (Roche et al. 2021).

Previous research on nursing staff's experiences of caring for persons diagnosed with AN in psychiatric inpatient care describes how staff often perceive their work as demanding regarding maintaining an empathetic therapeutic relationship and exercising professional authority to ensure adherence to treatment, which may create tensions in the relationship with the person with AN (Zugai et al. 2019). Staff in specialised eating disorder inpatient services have described emotional strain, relational tensions and the challenge of balancing support with treatment demands among staff caring for persons with AN (Bommen et al. 2023; Davén et al. 2022). Taken together, regardless of whether the care is provided within nonspecialised or specialised eating disorder inpatient services, staff describe nursing care for persons with AN as complex and demanding. However, less attention has been paid to how such tensions and practices are shaped by broader understandings of illness, care and professional responsibility within psychiatric inpatient contexts. Rather than treating these as separate issues, the present study approaches them as interconnected aspects of how care for persons with AN is understood and enacted within GPIC.

### 2.1 A Foucauldian Perspective on Psychiatric Care

Research suggests that care for persons with AN in GPIC is primarily grounded in a biomedical approach, emphasising measurable indicators such as weight restoration, food intake and laboratory results (Sandsten, Lindgren, et al. 2025). Such an approach can seem reductive as little attention is directed towards comorbidities or underlying causes of AN, making patients feel unseen as unique persons and potentially hindering their recovery process (Healey 2025; Moberg 2025; Rankin et al. 2023; Sandsten, Gabrielsson, et al. 2025). Thus, care for persons with AN in GPIC is not only organised around clinical needs but is also shaped by particular ways of understanding illness and responsibility. A Foucauldian perspective offers analytical tools for exploring the interplay of power, knowledge and subject positions in everyday psychiatric practice.

In Foucauldian terms (1982b), power is understood not as a possession or a solely repressive force but as relational and productive, operating through everyday practices that structure care. This means that power not only restricts action but also shapes what is considered appropriate to do, contributing to care logics in which certain practices come to appear necessary, responsible and professionally legitimate. Power and knowledge are mutually constitutive: certain ways of knowing and acting are rendered legitimate, while others are marginalised. In practice, this implies that clinical understandings, such as focus on weight and food, do not merely reflect the condition but also contribute to shaping how care is organised and what actions are prioritised. What comes to count as authoritative or ‘true’ is therefore approached as the effect of specific power–knowledge relations rather than as objective or universal truth (Foucault 1982a; Young 1987). In the context of GPIC, this implies that what comes to be understood as appropriate care, professional responsibility and necessary intervention is shaped through such power–knowledge relations, rather than being self‐evident or neutral.

From this standpoint, care practices, professional roles and forms of knowledge are not fixed or self‐evident but are constituted through historically and socially situated discourses (Foucault 1982a), that is, through shared ways of talking about and understanding illness and care. Discourses shape and are shaped by how illness, care and patients are made intelligible, as well as how professional responsibility comes to be recognised as legitimate. Furthermore, discourses may contribute to the production of care logics. In this study, care logic is used analytically to describe how discourses organise and justify care practices and professional responsibilities within GPIC. Subject positions refer to the roles and responsibilities made available to staff and patients in everyday GPIC practice, for example how nursing staff come to be positioned as responsible for managing risk and patients as requiring control or intervention. Such positions are not freely chosen but emerge within discursive arrangements that organise care in taken‐for‐granted ways, making some actions appear appropriate and legitimate while constraining others.

### 2.2 Aim

Critical perspectives on staff experiences can provide valuable insights to inform future research and mental health nursing practice in the care of persons with AN. Thus, the aim of the study was to explore how nursing staff experience and understand care for persons with AN within GPIC settings, with particular attention to the conditions shaping what comes to count as legitimate nursing practice.

## 3 Methods

Qualitative design was employed, using individual semi‐structured interviews subjected to abductive qualitative content analysis (Graneheim et al. 2017; Graneheim and Lundman 2004). An abductive approach was chosen to support the interpretative construction of themes grounded in the data, while enabling a theoretically informed analysis of the broader structural conditions shaping practice. A Foucauldian‐inspired theoretical perspective was considered suitable as the study aimed to explore not only experiences of care, but also how practices and responsibilities are shaped within broader discursive conditions. Authors one, two, four and five are nurses specialised in psychiatric care. The third author is a physiotherapist specialised in mental health with a background in gender studies. All authors have extensive experience of providing psychiatric care for complex psychiatric conditions and AN. This study is reported in accordance with the consolidated criteria for reporting qualitative research (COREQ) checklist (Tong et al. 2007).

### 3.1 Participants and Procedures

Participants were recruited in Sweden through social media, with assistance from a mental health interest group. Potential participants were invited to access an online form that provided detailed study information and enabled them to give informed consent. The inclusion criterion was to have experience in nursing care for persons with AN in GPIC, including child and adolescent psychiatry. The exclusion criterion was not being nursing staff.

Nine nursing staff with professional experience of working in GPIC units in different parts of Sweden participated in the study (Table 1).

**TABLE 1:** Overview of participant characteristics.

Characteristics	*n*/Range	Median
Registered nurses (including three specialist nurses)	6	
Assistant nurses	3	
Women/men	8/1	
Age (years)	29–53	43
GPIC experience (years)	1.5–16	6

Abbreviation: GPIC = general psychiatric inpatient care.

### 3.2 Interviews

One‐on‐one interviews were conducted by the first, second and last author, all with experience in qualitative interviewing. Participants were informed about the interviewer's professional background prior to the interview. In the case that a prior relationship existed between a participant and a member of the research team, another member of the research team conducted the interview. The interviews took place in March–October 2025. The authors developed an interview guide, consisting of open‐ended questions such as *How do you understand AN as an illness? How would you describe your role in* the *care of persons with AN?* and *How did you involve persons with AN in their own care and were there situations where this became difficult?* Follow‐up and clarifying questions were used to encourage participants to elaborate on their experiences and reflections. The interviews lasted between 42 and 72 (median 58) minutes and were audio recorded.

### 3.3 Qualitative Content Analysis

Transcribed interviews were read repeatedly to gain an overall understanding. The analysis followed an abductive approach. Initially, an inductive procedure was applied, involving identification of meaning units, condensation, coding, abstraction and interpretation into subthemes (Graneheim et al. 2017). For example, the codes ‘Trying to stay calm, reassure the patient, and at the same time go against their will takes a toll on you’, ‘Coercive measures must not violate the patient; failure feels like an infringement’ and ‘Consoling oneself that coercive measures are necessary to prevent death, and are medically justified’ were grouped into the subtheme *Normalising coercion in everyday care*. In the subsequent deductive phase, the subthemes were interpreted through a Foucauldian‐inspired lens, drawing on concepts of discourse, power and subject positioning (Foucault 1982b), resulting in two broader themes.

## 4 Findings

The findings illuminate how nursing staff in GPIC experience and understand AN and the provision of AN care are presented as two themes. Within each theme, the subthemes reflect the central patterns mediated in the interviews (Table 2).

**TABLE 2:** Overview of the subthemes and themes revealed during the analysis.

Subthemes	Themes
Making sense of AN through risk and stabilisation	Nursing practice governed by a risk‐oriented care logic
Enacting control through routines and treatment plans	
Normalising coercion in everyday care	
Navigating responsibility under unstable care conditions	Responsibility as a burden and a possibility within nursing practices
Producing alternative care practices through relational work	

## 5 Nursing Practice Governed by a Risk‐Oriented Care Logic

The first theme, *Nursing practice governed by a risk‐oriented care logic*, captures how participants experienced working within a care logic structured by control, standardisation and harm prevention. Participants frequently described AN as a life‐threatening and complex condition requiring control and structure. This understanding appeared closely intertwined with a focus on risk, responsibility and conflicting demands. Within this logic, care was described as organised around manuals, standardised routines and systematic risk assessments. Through these practices, coercive measures were enabled and gradually normalised in everyday care. Participants' descriptions suggest that routines and risk assessments did not merely regulate care but also shaped what came to be seen as appropriate and responsible action. The structuring of care practices through standardisation and risk management reflects how power operates productively (Foucault 2009), shaping a care logic in which control appears necessary, responsible and ethically justified. Hence, what might otherwise be regarded as intrusive or exceptional interventions came to be framed as necessary, positioning nursing staff as responsible subjects expected to enact control and prevent harm within everyday care practices. At the same time, participants experienced these practices as being at odds with nursing ethics and their conception of meaningful care, but also as difficult to contest within the prevailing care logic, as deviations could be interpreted as irresponsible or unsafe.

### 5.1 Making Sense of AN Through Risk and Stabilisation

Participants described AN as glamorised and normalised in society where thinness is socially valued even in the presence of severe illness. They described this societal framing as being linked to assumptions of AN as a self‐inflicted condition and to gendered norms positioning the illness as predominantly female. In relation to this, participants noted that most persons with AN in GPIC were women and suggested that this norm probably made help seeking particularly shameful for men and boys. They further argued that men and boys may instead manage psychological distress through other behaviours, such as substance use, violence or excessive exercise or they may develop less life‐threatening eating disorders less likely to require GPIC.

It's often perceived as a female illness. […] There is a societal view that it's more masculine to turn to substances than to starvation. (Participant 3)

Persons admitted to GPIC were often acutely ill, requiring an initial focus on stabilisation. According to the participants nursing staff initially often felt fear and uncertainty when caring for persons with AN. However, over time they developed a more nuanced understanding of the illness as a complex psychological condition linked to control, emotional regulation or self‐punishment, rather than appearance. This relational and experiential understanding contrasted with societal views and reinforced participants' sense that the structure of GPIC did not always accommodate the psychological depth of the illness.

I think it [AN] is very much about the patient having strategies for managing anxiety in some way. It's about thoughts and feelings and really, about life itself, but where you somehow control your existence through food. (Participant 7)

Participants described how caring was organised around one diagnosis at a time, with acute somatic needs typically taking precedence. Comorbidities, such as depression, trauma or neurodevelopmental conditions, were described as pervasive yet organised into hierarchical trajectories where the need considered most acute shaped the overall course of care. This approach often left staff uncertain about how to respond to interacting needs, particularly, in situations involving self‐harm, intense anxiety or pronounced need for control, which were described as common and difficult to manage.

There is this idea that you have to address one thing at a time. You must gain weight first to maybe receive trauma treatment or talking therapy or undergo an assessment. But many of the patients I've met have some form of trauma as well. You can't just treat the low weight; you must see the bigger picture. (Participant 10)

Participants described experiencing uncertainty, particularly, regarding autism and trauma and linked this uncertainty to variations in knowledge and competence among staff. While comorbidity was recognised as requiring broad competence and individualised adaptations, uncertainty about how to support overlapping needs often led staff to narrow their focus to a single diagnosis. Participants described this narrowed focus as resulting in inadequate care and an increased risk of conflict and the use of coercive measures.

What I found most difficult was knowing what the focus should be when there were several things going on simultaneously. Often, if nothing alarming showed up in the blood samples, the focus shifted toward depression or anxiety instead. (Participant 9)

Participants described a recurring sense of powerlessness when caring for persons with a complex illness, where often their role was reduced to keeping the person with AN alive. This gave rise to frustration, sadness and uncertainty, particularly when no improvement was perceived. Conversations about the body and appearance, as well as encounters with intense anxiety, anger or silence were described as especially challenging, as participants often felt unsure how to help.

### 5.2 Enacting Control Through Routines and Treatment Plans

Participants described GPIC as organised around food intake and weight, with limited knowledge and routines for relational approaches. They portrayed care as rigid and standardised, structured through predefined treatment and BMI‐directed meal plans assumed to fit everyone despite variation in the underlying causes of AN. This emphasis on control was experienced as limiting flexibility in nursing care. It took attention away from the person behind the measurements, as adherence to plans took precedence over responding to individual needs.

I think staff in inpatient care are afraid to deviate from the treatment plan and that can make things very rigid … […] I think it's a fear of doing something wrong, of causing consequences for the patient and maybe for oneself as a staff member too. You want to do what has been decided. You want to do the right thing. (Participant 6)

Participants perceived that staff often shared understandings of AN centred on manipulation and the need for consistency, which guided everyday care decisions. These understandings led staff to prioritise uniformity and adherence to routines, limiting space for participation by the person in care and thereby potentially intensifying anxiety and reinforcing existing power imbalances rather than alleviating suffering.

I experience the focus as being on limiting and controlling on preventing compensatory behaviors and getting the patient to comply with the meal plan—but perhaps with less attention to the psychological aspects and what this actually evokes in the patient. (Participant 3)

Some tailoring of care and personal adaptations did occur; however, they were limited and situational. Suggestions by a person with AN often required in‐team negotiation and were retrospectively judged against weight outcomes. If there was a decrease in weight, the suggestion was considered unsuccessful.

### 5.3 Normalising Coercion in Everyday Care

Some participants described coercive measures as a necessary part of care, directed at the illness rather than the person. However, care was also described as characterised by an underlying threat of coercion, as non‐adherence to food or weight plans could lead to consequences such as tube feeding or withdrawal of activities. In some cases, responsibility for coercive measures was described as shifting to parents because of unclear boundaries regarding parental consent for minors

If the patient does not manage to take in the required nutrition and ends up with a deficit. […] there is an opportunity to compensate, but if the person in care can't manage that, it may lead to tube feeding. (Participant 4)

Participants described coerced tube feeding in GPIC as routine and not always limited to acute situations. Repeated insertion and removal of feeding tubes, often combined with restraint, was experienced as distressing and as reinforcing power struggles. Whether medically necessary, these measures were perceived as violations and as potentially traumatising, creating fear through the constant threat of coercion.

It was decided that if the patient didn't finish the meals, everything would be added up to meal by meal and given through a feeding tube later in the evening. And I didn't really feel that it was right. Especially since the order was also that the tube should be removed after each occasion, meaning it had to be reinserted every time. And we knew that this person would need to compensate in some way every single day. (Participant 2)

The use of coercive measures was described as emotionally demanding and ethically troubling for staff, even when carried out in accordance with routines. Acting against another person's will, witnessing suffering and recognising the risk of trauma generated ethical distress. To manage this, participants described justifying their actions by framing coercion as life‐saving and grounded in physicians' medical assessments. Some also described a view that patients might later express gratitude, an understanding that helped staff remain convinced they were acting in the patient's best interest.

Imagine really not wanting to have the feeding tube inserted and then we push the tube down the nose and administer the liquid, and they are so overwhelmed by anxiety. They think, ‘Now I'm going to die.’ I mean, I think that's … just imagine that feeling … (Participant 9)

Participants also described limited knowledge among staff regarding strategies to prevent coercion. Situations were perceived as more likely to escalate when staff did not trust the person in care, failed to take them seriously or did not create opportunities for participation. Misinterpretations of expressions from persons with AN, as well as responses to anxiety characterised by irritation rather than understanding, were described as particularly problematic. Across accounts, participants expressed the view that many coercive situations could have been avoided through alternative approaches to care, which they believed might have reduced fear of coercion among persons receiving GPIC.

There is this profound suffering and it's as if they are constantly being exposed to exactly what they are most afraid of, sometimes in rather insensitive ways. And I don't think that this is always understood as fear and anxiety. Instead, I think staff often perceive it as them being… well, quite combative. (Participant 8)

## 6 Responsibility as a Burden and a Possibility Within Nursing Practices

The second theme, *Responsibility as a burden and a possibility within nursing practices*, highlights how responsibility was shaped within organisationally unstable care conditions, where maintaining safety, setting boundaries and adhering to routines often became central markers of what it meant to act as a responsible nurse. At the same time, participants described how relational engagement and personal judgement occasionally opened space for more flexible and collaborative nursing care. Participants described how they were expected to carry responsibility for preventing harm and maintaining control despite unstable organisational conditions in terms of staffing, insufficient knowledge and shifting care priorities. At the same time, responsibility could be understood as a possibility when it created space to find new ways forward in care together with the person in care. Responsibility may however be understood not merely as a personal attribute, but as shaped within a risk‐oriented care logic where maintaining safety, control and adherence to routines became central to what was understood as responsible nursing practice. However, in navigating everyday practice, participants described how routines and general recommendations were at times stretched and reinterpreted, opening limited space for care shaped through relational negotiation. In these moments, the person with AN was not only constituted as an object of intervention but also engaged as a participant in shaping care.

### 6.1 Navigating Responsibility Under Unstable Care Conditions

Participants described GPIC as organisationally unstable, characterised by high staff turnover and fluctuating medical presence. Variations in physicians' knowledge of and engagement in AN were experienced as undermining continuity of care and contributing to shifting priorities over time. Limited introduction and minimal emphasis on psychiatric nursing in education left participants feeling insufficiently prepared to care for persons with AN, reinforcing a sense of professional inadequacy and a need for support in managing complex situations and challenging conversations.

Some physicians are very open to dialogue and communication about what the patient wants […] Others may not really have sufficient knowledge or understanding of how AN works. In those cases, it can become more about this kind of educating approach, where boundaries are set and the patient is expected to understand that they must eat and do things in a certain way. (Participant 3)

Care was described as highly dependent on individual staff members' knowledge, experience and personal approach, resulting in considerable variation in how care was delivered. While participants found this reliance on relational skills and personal judgement as demanding, they also described a strong sense of meaning when their presence appeared to make a difference for the person in care. Professional roles were perceived as shaping different degrees of distance and influence in care, affecting how responsibility and relational engagement were enacted.

They [nurses] often just hand out medications and walk around in the ward. Sometimes they might stop by and say hello. Sometimes they might have a brief conversation with the patient, but not in the same way as we nursing assistants do. (Participant 4)

Participants described how although AN was not the most common reason for admission in GPIC, there was often at least one person with AN hospitalised for prolonged periods, frequently under coercion. This created conditions for close relationships, while care was simultaneously described as predominantly short‐term and reactive, leaving limited space for structured nursing work or the development of a sustainable care environment. The everyday environment in GPIC was experienced as chaotic, with meals described as particularly vulnerable situations, as comments from others increased anxiety, while eating in isolation risked reinforcing withdrawal. Participants further described how the time between meals was largely unstructured and lacking meaningful content for persons with AN and suggested that this time could instead be used for activities perceived as supporting recovery and helping persons with AN reconnect with everyday life beyond GPIC.

### 6.2 Producing Alternative Care Practices Through Relational Work

Participants described how weight gain in persons with AN was rarely the most challenging aspect of care; rather, the main difficulty lay in providing long‐term support. Many persons with AN were described as being discharged with a normalised BMI but with persistent inner conflict and anxiety. Participants emphasised the need for nursing care to address anxiety management and AN‐driven thoughts during GPIC and described how a stronger nursing perspective was perceived as better able to support personal recovery.

Yes, they eat while they're in the ward, but then, when they are discharged, what happens? They are expected to live with this new body, with the new weight, and that involves immense suffering. And they may also develop a misleading idea of what it means to be well. They think, ‘Now I'm healthy because I have a normal BMI,’ while the same battle is still going on inside their heads. (Participant 3)

Relational work was experienced as central to care, with time spent building relationships, fostering trust and openness and enabling more accurate assessments of needs. At the same time, relational work was experienced as more demanding than following standardised protocols. Participants described variation in the leeway they had to adapt care by challenging routines or medical decisions, with experience contributing to increased confidence in leading nursing care and in testing patient suggestions or offering care alternatives.

Once you've worked a bit longer you dare to make your own decisions. […] you dare to find alternative solutions and do it in consultation with the patient, rather than just following the physician's orders and care plans. And that was when I realized that you might start to reach these patients when you dare to be a bit less rigid. (Participant 10)

## 7 Discussion

In our findings, nursing care in GPIC was often described as a rational and clinically necessary response to a potentially life‐threatening illness, organised around stabilisation, risk management and acute medical priorities. Such understandings reflect a discourse in which AN is primarily constituted as a condition of bodily risk requiring control, surveillance and intervention. When these understandings repeatedly become the basis for judging good care, they also shape what is recognised as appropriate and professionally responsible practice. From a Foucauldian‐inspired perspective (Foucault 1981), this suggests that nursing practices are organised through a care logic that stabilises particular ways of understanding illness, responsibility and legitimate intervention. The challenges described by participants can thus be understood not as personal failures or deficits of competence but as effects of working within a care logic that stabilises particular ways of knowing and acting. As Eivergård et al. (2021) argue, professional language does not merely reflect care priorities but participates in constituting the subject positions through which those priorities become self‐evident. In practice, this means that ways of talking about care, for example in terms of risk, stabilisation and adherence, also shape how staff understand their role and what actions come to be seen as appropriate and responsible.

This care logic, articulated through control, manuals and a prevailing risk orientation, aligns professional responsibility with adherence to predefined routines and treatment plans. A risk‐oriented understanding shapes what is recognised as legitimate care, rendering adherence to established plans self‐evident and framing coercive measures as necessary responses to risk (Foucault 1981). As illustrated in participants' descriptions of a fear of deviating from the treatment plan and of ‘not doing the right thing’, nursing staff appeared to position themselves as responsible for maintaining safety and preventing harm through adherence to established routines and recommendations. Power thus operates not primarily through overt coercion of staff, but through the internalisation of accountability and self‐regulation. These descriptions of responsibility, often expressed in terms of needing to do the right thing and avoid deviation, further point to how a risk‐oriented care logic shapes what comes to count as responsible practice. This aligns with Foucauldian analyses demonstrating how risk discourses organise psychiatric care and produce accountable professional subjects (Bakke et al. 2025).

In our findings, professional responsibility emerges as both a burden and a possibility as staff navigate demands that are differently valued within prevailing care logic. These tensions can be understood as effects of how particular knowledge is authorised and stabilised within everyday care, while others are rendered less visible or less defensible (Foucault 1981). Professional responsibility thus becomes both a source of meaning and a source of strain as nursing staff navigate differently valued demands while remaining accountable to dominant norms of legitimate care. Importantly, this dynamic should not be understood as reflecting a lack of commitment to relational or personalised care. Rather, it highlights how relational nursing work often needs to be articulated through biomedical terms to be recognised as legitimate.

Previous research has described nurses' experiences of emotional strain, being an authority and relational challenge in inpatient eating disorder care (Davén et al. 2022; Zugai et al. 2019). The present study extends this work by examining how such experiences are shaped within a care logic that defines what becomes possible and defensible as professional responsibility. In participants' descriptions, this was reflected in how staff often needed to justify care decisions in relation to prevailing clinical priorities, such as weight restoration, stabilisation or risk reduction. Using a Foucauldian lens (Foucault 1982a), professional responsibility thus becomes oriented not only towards caring for the person with AN but also towards ensuring that care can be rendered intelligible and defensible within prevailing regimes of knowledge, for example, through its contribution to stabilisation, weight gain or risk reduction. This illustrates how power operates by shaping the conditions under which care is recognised as legitimate and enacted as professional responsibility.

These findings further illuminate the participants' experiences of how relational work at times enabled care practices to be configured differently within prevailing care logic. In the second theme, participants described situations where routines were approached through listening, adaptation or the creation of relational space, thereby expanding what could count as legitimate care in specific encounters. These moments of adaptation suggest that prevailing routines were not fixed but open to negotiation in practice. From a Foucauldian perspective, such reconfigurations can be understood as situated effects of how professional legitimacy and scope of action are negotiated and stabilised within prevailing care logic (Foucault 1981). Although enacted by individual nursing staff, such practices should not be understood as personal solutions but as contingent practices emerging from the interplay between professional positioning and shifting conditions for legitimate action.

Taken together, the findings suggest that risk‐oriented routines and reasoning function as primary means through which professional legitimacy is secured in GPIC. While relational nursing practices were present, they remained contingent, unevenly supported and difficult to sustain as legitimate care. As a result, care for persons with AN was most often organised around what could be defended within this care logic, rather than around relationally negotiated understandings of what was considered sufficient in specific encounters.

## 8 Study Limitations

This qualitative, theory‐informed study aims for analytically grounded insights into how nursing staff's experiences of caring for persons with AN are shaped and interpreted within GPIC. Its value lies in rendering visible the conditions that shape how care is enacted and recognised as professional responsibility. The scope, recruitment strategy and sensitive topic likely influenced which experiences were expressed, potentially limiting diversity and leading some aspects of practice to be underexplored or described more neutrally.

The theoretical perspective does not claim to represent participants' own framings but offers one way of situating the findings within broader discursive conditions. Separating the analytic procedures reduces the risk of imposing theory prematurely, while making it explicit that the discussion reflects a theoretically informed interpretation. Prior knowledge sensitised the abductive analysis, while alternative interpretations were systematically discussed within the research team to challenge preliminary readings and refine themes. Interpretations were continuously checked against the data.

## 9 Conclusion

This study illustrates how nursing care for persons with AN in GPIC becomes organised through a discourse centred on risk, stabilisation and harm prevention. A discourse giving rise to a prevailing care logic in which adherence to routines and treatment plans comes to signify professionally responsible care. Within this care logic, nursing staff are positioned as responsible subjects, where maintaining control and adhering to manuals become constructed as accountable nursing practice, while relational and individualised nursing practices become more difficult to sustain and justify. At the same time, participants described moments where routines were negotiated and care practices adapted through relational engagement, suggesting that this care logic was neither fixed nor total. By shifting attention from individual staff members to the broader conditions shaping practice, the study highlights how institutional understandings of risk, accountability and appropriate intervention influence what becomes possible, legitimate and recognisable as nursing care in GPIC.

## 10 Relevance for Clinical Practice

The findings suggest that the central issue is not the availability of nursing competence, but the conditions under which such competence can be enacted and recognised within the prevailing care logic. This means that difficulties in care may not solely reflect the complexity of AN itself, but also how care is organised and what forms of practice are institutionally supported. The study highlights the importance of critically examining governing ways of understanding AN and inpatient care, which shape what is considered legitimate nursing practice. The study offers a perspective for reflecting on why relational nursing work, individualised adaptations and ethical concerns may be experienced as difficult to sustain in everyday practice, despite being recognised as clinically meaningful. Such critical awareness can support team‐based reflection on how routines, risk reasoning and documentation practices shape professional responsibility and the use of coercive measures, without reducing these challenges to individual‐level explanations. The study suggests that improving care may require reflection on the care conditions that determine which knowledge can be used in practice.

## Author Contributions

**Anna Sandsten:** conception and design of the study, data collection, transcription of interviews, analysis, writing of original draft, review and revisions of subsequent drafts, correspondence with the publisher and approved final draft. **Maria Strömbäck:** conception and design of the study, data collection, feedback throughout the analysis process, reflective and critical approach throughout the research process, input across multiple drafts, review and editing and approved final draft. **Sebastian Gabrielsson:** conception and design of the study, feedback throughout the analysis process, input across multiple drafts, review and editing and approved final draft. **Git‐Marie Ejneborn Looi:** conception and design of the study, feedback throughout the analysis process, input across multiple drafts, review and editing and approved final draft. **Britt‐Marie Lindgren:** conception and design of the study, data collection, feedback throughout the analysis process, input across multiple drafts, review and editing, correspondence with the publisher and approved final draft. All authors meet the authorship criteria according to the latest guidelines of the International Committee of Medical Journal Editors.

## Funding

The authors have nothing to report.

## Ethics Statement

The study received ethical approval from the Swedish Ethical Review Authority (Dnr 2022‐05985‐01; 2024‐05404‐02) and was conducted in accordance with the ethical guidelines described in the World Medical Association (2013).

## Consent

The participants were informed about the purpose of the study, the voluntary nature of their participation and their right to withdraw from the study at any time without explanation. All participants gave their informed consent to participate.

## Conflicts of Interest

The authors declare no conflicts of interest.

## Supporting information


**Data S1:** COREQ checklist.

## Data Availability

The data that support the findings of this study are not publicly available due to the sensitive nature of the data and the risk of participant identification but may be available from the corresponding author upon reasonable request.
