# Supplementary material for: Professional Responsibility Governed by a Risk‐Oriented Care Logic: Nursing Staff's Experiences of Caring for Persons With Anorexia Nervosa in General Psychiatric Inpatient Care
Source: Int J Ment Health Nurs. 2026 Jun 22;35(3):e70297. doi: 10.1111/inm.70297 (PMC13284724; doi:10.1111/inm.70297)
Supplement: Supplementary file 1 — Data S1: COREQ checklist. [file INM-35-0-s001.docx]

| No | Item | Guide question | Reported on page |
| --- | --- | --- | --- |
| Domain 1: research team and reflexivity | | | |
| *Personal characteristics* | | | |
| 1 | Interviewer/facilitator | Which author/s conducted the interview or focus group?  *AS, MS & BML* | 5, 14-15 |
| 2 | Credentials | What were the researcher’s credentials? E.g. PhD, MD | 1 |
| 3 | Occupation | What was their occupation at the time of the study? | 1 |
| 4 | Gender | Was the researcher male or female?  *The research group consists of both men and women.* | 1 |
| 5 | Experience and training | What experience or training did the researcher have? | 1 |
| *relationship with participants* | | | |
| 6 | Relationship established | Was a relationship established prior to study commencement?  *No prior relationship was established with most participants. In cases where a prior acquaintance existed, participants were offered to be interviewed by another member of the research team.* | 6 |
| 7 | Participant knowledge of the interviewer | What did the participants know about the researcher?  *The interviewers introduced themself and their background prior to the interviews via e-mail.* | 6 |
| 8 | Interviewer characteristics | What characteristics were reported about the interviewer/facilitator? | 1, 13 |
| domain 2: study design | | | |
| *theoretical framework* | | | |
| 9 | Methodological orientation and theory | What methodological orientation was stated to underpin the study? | 2-5 |
| *Participant selection* | | | |
| 10 | Sampling | How were participants selected? | 4 |
| 11 | Method of approach | How were participants approached? | 4 |
| 12 | Sample size | How many participants were in the study? | 4 |
| 13 | Non-participation | How many people refused to participate or dropped out? Reasons? | N/A |
| *Setting* | | | |
| 14 | Setting of data collection | Where was the data collected? | 5 |
| 15 | Presence of non-participants | Was anyone else present besides the participants and researchers?  *No.* | N/A |
| 16 | Description of sample | What are the important characteristics of the sample? | 5 |
| *data collection* | | | |
| 17 | Interview guide | Were questions, prompts, guides provided by the authors? Was it pilot tested?  *The guide was not pilot tested prior to data collection.* | 5 |
| 18 | Repeat interviews | Were repeat interviews carried out? If yes, how many?  *No.* | N/A |
| 19 | Audio/visual recording | Did the research use audio or visual recording to collect the data?  *Audio.* | 5 |
| 20 | Field notes | Were field notes made during and/or after the interview or focus group?  *No.* | N/A |
| 21 | Duration | What was the duration of the interviews or focus group? | 5 |
| 22 | Data saturation | Was data saturation discussed?  *Yes, during the analyzing process. The interviews were extensive and in-depth, indicating that data saturation had been achieved.* | N/A |
| 23 | Transcripts returned | Were transcripts returned to participants for comment and/or correction?  *No.* | N/A |
| domain 3: analysis and findings | | | |
| *Data analysis* | | | |
| 24 | Number of data coders | How many data coders coded the data? | 13 |
| 25 | Description of the coding tree | Did authors provide a description of the coding tree? | 5, 6 |
| 26 | Derivation of themes | Were themes identified in advance or derived from the data? | 4-5 |
| 27 | Software | What software, if applicable, was used to manage the data?  *No.* | N/A |
| 28 | Participant checking | Did participants provide feedback on the findings?  *No.* | N/A |
| *reporting* | | | |
| 29 | Quotations presented | Were participant quotations presented to illustrate the themes / findings? Was each quotation identified?  *Each quotation was identified by participant number.* | 6-11 |
| 30 | Data and findings consistent | Was there consistency between the data presented and the findings? | 6-11 |
| 31 | Clarity of major themes | Were major themes clearly presented in the findings? | 5-9 |
| 32 | Clarity of minor themes | Is there a description of  diverse cases or discussion of minor themes? | 5-11 |
